# Transmission of Avian Influenza Virus (H3N2) to Dogs

**DOI:** 10.3201/eid1405.071471

**Published:** 2008-05

**Authors:** Daesub Song, Bokyu Kang, Chulseung Lee, Kwonil Jung, Gunwoo Ha, Dongseok Kang, Seongjun Park, Bongkyun Park, Jinsik Oh

**Affiliations:** * Green Cross Veterinary Products Company, Ltd., Yong-in, South Korea; † Daewoong Pharmaceutical Company, Ltd., Yong-in, South Korea; ‡ Animal Genetics, Inc., Suwon, South Korea; § Seoul National University, Seoul, South Korea; 1 These authors contributed equally to this article.

**Keywords:** influenza virus, H3N2, dog, avian, research

## Abstract

In South Korea, where avian influenza virus subtypes H3N2, H5N1, H6N1, and H9N2 circulate or have been detected, 3 genetically similar canine influenza virus (H3N2) strains of avian origin (A/canine/Korea/01/2007, A/canine/Korea/02/2007, and A/canine/Korea/03/2007) were isolated from dogs exhibiting severe respiratory disease. To determine whether the novel canine influenza virus of avian origin was transmitted among dogs, we experimentally infected beagles with this influenza virus (H3N2) isolate. The beagles shed virus through nasal excretion, seroconverted, and became ill with severe necrotizing tracheobronchitis and bronchioalveolitis with accompanying clinical signs (e.g., high fever). Consistent with histologic observation of lung lesions, large amounts of avian influenza virus binding receptor (SAα 2,3-gal) were identified in canine tracheal, bronchial, and bronchiolar epithelial cells, which suggests potential for direct transmission of avian influenza virus (H3N2) from poultry to dogs. Our data provide evidence that dogs may play a role in interspecies transmission and spread of influenza virus.

## Abstract 2

Infection with and transmission of the virus was identified in pet dogs with severe respiratory disease.

*Influenza A virus,* a member of the genus *Orthomyxovirus,* is an economically important virus that causes disease in humans, pigs, horses, and fowl (*1*). A crucial feature in the ecology and epidemiology of influenza virus is interspecies transmission (*2*). The emergence of new virus subtypes and their interspecies transmission is of great concern; measures to counteract their spread are vital for preventing influenza epidemics and pandemics. One of the basic mechanisms of interspecies transmission of influenza virus is direct transfer of an essentially unaltered virus from 1 species to another (*3*); however, some factors restrict this transfer. In particular, the presence or absence of host species–specific influenza virus binding receptors in the upper and lower respiratory tracts serves to prevent such cross-species or zoonotic transmission. Human influenza viruses bind to glycolipids or glycans that contain terminal sialyl-galactosyl residues with α 2,6-gal linkages (SAα 2,6-gal), whereas avian influenza viruses bind to residues with SAα 2,3-gal linkages (*4*). Examples of interspecies transmission of influenza viruses include recent human infections with the H5N1 subtype of avian influenza virus, and in canine infections with the H3N8 subtype of equine influenza virus (*3*,*5*). In addition, influenza infections were recently reported in species (canine, feline) that historically do not carry influenza virus (*6*). However, most directly transmitted infections of entire influenza viruses from a natural host species to a new host species do not result in sustained transmission in the new host species (*3*). Therefore, establishing new, long-lived influenza virus lineage is uncommon and difficult (*7*).

We report interspecies transmission of a complete avian influenza virus (H3N2) to dogs and the emergence of a new canine influenza virus associated with acute respiratory disease in South Korea, where avian influenza viruses (H3N2, H5N1, H6N1, and H9N2) currently circulate or have been previously detected (*8*). We investigated pathogenicity of the isolated virus in experimental dogs and evaluated localization of SAα 2,6-gal and SAα 2,3-gal linkages in upper and lower canine respiratory tracts.

## Materials and Methods

### Outbreak Histories

From May through September 2007, cases of severe respiratory disease occurred in animals at 3 veterinary clinics located 10–30 km apart in Kyunggi Province and 1 kennel located in Jeolla Province (southern part of South Korea). The first case, which occurred in May, was identified in a 5-year-old miniature schnauzer that had nasal discharge for 3 days and sneezing for 2 days, after which the signs subsided and the dog recovered. In August, another case was identified in a 3-year-old cocker spaniel that had fever, cough, nasal discharge, and anorexia and died after the onset of clinical signs. In September, severe respiratory disease was identified in 2 Jindo dogs (a Korean breed of hunting dog that originated on Jindo Island) and a 3-year-old Yorkshire terrier. These animals had severe cough, fever, and nasal discharge and died 2 days after visiting the same animal hospital. Finally, an outbreak of canine influenza occurred in an animal clinic in which all 13 dogs housed in a shelter facility were found to be infected with the same virus; their clinical signs were nasal discharge, cough, and high fever. Of the dogs in the affected kennel in Jeolla Province, paired serum samples showed that 47 (90%) of 52 were seropositive for canine influenza virus (H3N2) at the first sampling and that 100% were seropositive by the second sampling.

Nasal swabs from the miniature schnauzer, cocker spaniel, and Yorkshire terrier were submitted to Animal Genetics, Inc. (Suwon, South Korea) for reverse transcription–PCR (RT-PCR) and testing with a commercial rapid influenza virus antigen detection kit (Animal Genetics, Inc.). Hemagglutinin inhibition (HI) tests were performed according to the World Organization for Animal Health recommendations; commercial nucleocapsid protein (NP)–based ELISA (Animal Genetics, Inc.) was used for serologic testing.

### RT-PCR and Sequencing

Nasal swabs from the above-mentioned 3 dogs were also used to isolate the influenza A virus by inoculation into 11-day-old chicken eggs. After 3–4 days of incubation, allantoic fluids were clarified by low speed centrifugation, and these fluids were shown to agglutinate chicken erythrocytes. Virus RNA was extracted from allantoic fluids by using Trizol LS (Molecular Research Center, Inc., Cincinnati, OH, USA) according to the manufacturer’s instructions. RT-PCR was performed under standard conditions with random hexamer primers. Isolated influenza virus was subtyped by RT-PCR analysis by using primers specific for canine, swine, and avian hemagglutinin 3 (H3) genes. Primers for the detection of viral genes H3, neuraminidase 2 (N2), polymerase basic protein (PB) 1, PB2, polymerase acidic protein (PA), NP, matrix protein (M), and nonstructural protein (NS) were designed by using the Primer 3 program with modifications (Whitehead Institute, Massachusetts Institute for Technology Center for Genome Research, Boston, MA, USA).

For PCR, pairs of primers were used to detect target genes. cDNA (2 μL) was mixed with a reaction mixture containing 2.5 μL of 10× Taq DNA polymerase buffer, 1.5 mmol/L MgCl_2_, 2.0 μL of dNTPs (2.5 mmol/L/μL), 1 μL of each specific primer (10 pmol/L each), and 1 μL of Taq DNA polymerase (Promega, Madison, WI, USA). Distilled water was added to make a final volume of 25 μL. PCR was performed by reaction initiation at 94°C for 10 min, amplification for 32 cycles at 94°C for 30 s, 55°C for 30 s, and 72°C for 30 s, and by final extension at 72°C for 10 min. The reaction was held at 4°C until further use. PCR products were analyzed by electrophoresis in 1.5% agarose gel containing ethidium bromide. Sequences of the isolated virus genes were edited and analyzed by using Bioedit software (www.mbio.ncsu.edu/BioEdit/bioedit.html). Phylogenetic trees were generated by using the MEGALIGN program (DNASTAR, Madison, WI, USA) with the ClustalX alignment algorithm (www.megasoftware.net).

### Experimental Infection with Isolated Virus

We experimentally reproduced viral infection in 10-week-old conventional beagle puppies that had been divided into inoculated (I) and noninoculated (NI) groups. Group I puppies (n = 9) were inoculated intranasally with 2 mL of virus isolate with a titer of 10^6.9^ 50% egg infectious dose (EID_50_)/0.1 mL; group NI puppies (n = 6) were inoculated intranasally with 2 mL of sterile phosphate buffered saline. Before they were inoculated, the animals were sedated by intramuscular injection of 0.1 mg/kg acepromazine malate (Bayer, Seoul, South Korea). Clinical signs of infection were monitored for 7 days after inoculation, and feces and nasal discharge were examined for virus shedding by RT-PCR for 10 days after inoculation. To detect antibodies against nucleoprotein and HI for hemagglutinin, we analyzed convalescent-phase serum samples from 3 puppies in each group for virus-specific antibodies by ELISA (Animal Genetics, Inc.). HI tests were performed according to World Organization for Animal Health–recommended procedures (*9*). At 3, 6, and 9 days postinoculation (dpi), 3 group I puppies and 2 group NI puppies were humanely euthanized for gross and histopathologic examination. All necropsy procedures were performed by veterinary pathologists. All organs from dogs and pigs (positive control) were rapidly immersed in 10% neutral formalin buffer to prevent autolysis and stored overnight. To detect influenza A virus antigens in group I or group NI tissues, we performed immunohistochemical examination by using goat anti–influenza A virus antibody (1:100; Chemicon, Temecula, CA, USA). To determine the presence or absence of SA α2,3-gal linkages comprising avian influenza virus receptors and SA α2,6-gal linkages comprising human influenza receptors in the respiratory tracts of noninfected puppies, lectin-based staining was performed as previously reported (*10*). Porcine tissue served as a positive control. All experimental procedures were approved by an independent animal care and use committee, and the guidelines of National Veterinary Research and Quarantine Service for the reproduction of pathogenesis in dogs were respected.

## Results

### Isolation of Virus

Nasal swabs from the miniature schnauzer, cocker spaniel, and Yorkshire terrier were positive for influenza virus and negative for other pathogens, including canine distemper virus, canine parainfluenza-2 virus, and *Bordetella bronchiseptica*. The isolated viruses were designated A/canine/Korea/01/2007 (H3N2), A/canine/Korea/02/2007 (H3N2), and A/canine/Korea/03/2007 (H3N2).

### Nucleotide Sequences

Eight gene segments (H3, N2, PB1, PB2, PA, NP, M and NS) of each isolated canine influenza virus were sequenced (EU127500, H gene; EU127501, N gene), and homologous sequences were sought in GenBank (Table). Sequences from avian influenza viruses that displayed homologies from 95.5% to 98.9% were identified for all 8 gene segments from 1 of the 3 subtype H3N2 canine isolates (A/canine/Korea/01/2007). The HA and NA genes of this isolate showed greatest identity with those of Korean avian influenza virus isolate S11, and the NS gene showed greatest identity to that of avian influenza virus (A/chicken/Nanchang/7-010/2000 [H3N6]) isolated from Chinese chickens. All the other genes, including PB1, PB2, PA, NP and M, were closely related to those of avian influenza virus isolated from ducks in Hong Kong, Japan, and China.

**Table:** Homology of the genes of A/canine/Korea/01/2007 influenza virus (H3N2) isolated in South Korea with related sequences in GenBank*

Gene†	Virus with the highest identity	Source	Identity, %	Accession no.
HA	A/chicken/Korea/S6/2003 (H3N2)	Avian	96.6	AY862607
NA	A/dove/Korea/S11/2003 (H3N2)	Avian	97.4	AY862644
PB1	A/duck/Yangzhou/02/2005 (H8N4)	Avian	98.9	EF061124
PB2	A/duck/Zhejiang/11/2000 (H5N1)	Avian	97.6	AY585523
PA	A/duck/Hokkaido/120/2001 (H6N2)	Avian	95.9	AB286878
NP	A/duck/Hong Kong/Y439/97 (H9N2)	Avian	95.5	AF156406
M	A/duck/Jiang Xi/1850/2005 (H5N2)	Avian	97.5	EF597295
NS	A/chicken/Nanchang/7-010/2000 (H3N6)	Avian	97.5	AY180648

*Influenza virus lineage of all RNA segments is avian. †HA, hemagglutinin, NA, neuraminidase; PB, polymerase basic protein; PA, polymerase acidic protein; NP, nucleocapsid protein; M, matrix protein, NS, nonstructural protein.

### Phylogenetic Relationships

**Figure 1:**
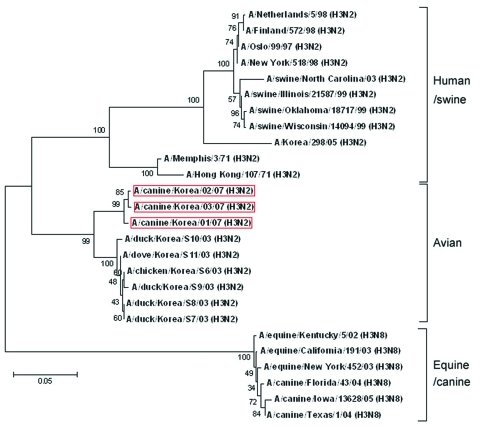
Phylogenetic relationship among hemagglutinin genes of canine influenza virus isolates. Tree of hemagglutinin genes from representative isolates from dog, human, bird, pig, and horse. Scale bar represents a difference of 5%. Red boxes indicate strains isolated in this study.

Phylogenetic analysis indicated that the canine influenza virus isolates from South Korea belonged to a different cluster than those of equine and canine influenza subtype H3N8 viruses. The HA and NA genes of the canine isolate (A/canine/Korea/01/2007 [H3N2]) were closely related to those of avian influenza virus (H3N2) from South Korea (Figure 1).

### Serologic Responses to Inoculation

All group I puppies had negative serologic assay results before inoculation. Group NI control puppies remained negative throughout the experiment.

**Figure 2:**
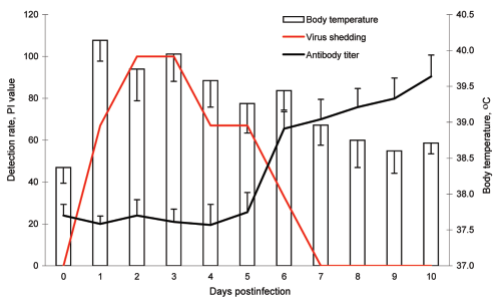
Body temperature, virus shedding, and antibody seroconversion after challenge with canine influenza virus. Body temperature was increased from 1 day postinoculation (dpi) and slowly decreased to normal temperature by 7 dpi. Virus shedding was detected from 1 dpi to 6 dpi by reverse transcription–PCR. However, the ELISA antibody titers increased after 6 dpi. Antibody titers were regarded as positive if percent inhibition (PI) was >50.

In nucleoprotein-specific ELISA, the percent inhibition values for group I at 6 dpi were substantially higher than those for group NI (Figure 2); and the HI antibody titers of group I (HI titer 80) were induced at 8 dpi.

### Clinical Responses to Challenge

Clinical signs, including sneezing and nasal discharge in group I, were observed at 2–7 dpi. The rectal temperatures of group NI animals remained below 39°C throughout the experiment. At 24 h after inoculation, fever developed in group I puppies (mean rectal temperature 40.14°C) (Figure 2) and lasted through 6 dpi.

### Virus Shedding

Influenza virus was not detected in feces. However, for group I puppies, virus shedding in nasal discharge began at 1 dpi and continued to 6 dpi; the highest titers, 10^6.1^(EID_50_/0.1 mL), were reached by 4 dpi. RT-PCR products generated from shed viruses were sequenced and identified as identical to the inoculated virus.

### Histopathologic Findings

**Figure 3:**
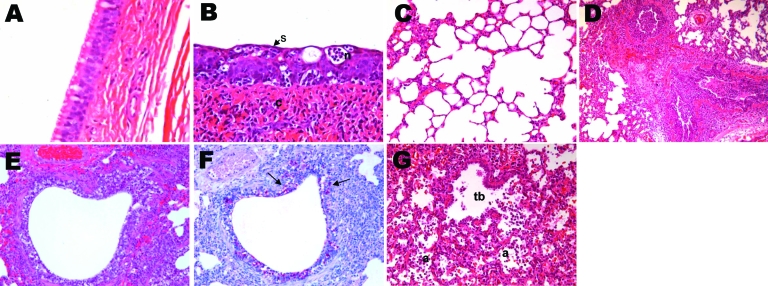
Histopathologic lesions in the trachea and lungs of control (A and C) or experimentally infected (B, D–F) dogs (A/canine/Korea/01/2007 [H3N2]) at different days postinoculation (dpi). A) Control dog at 9 dpi, showing normal pseudostratified columnar epithelium lining of the trachea; original magnification ×400. Hematoxylin and eosin (HE) stain. B) Influenza-infected dog at 9 dpi, showing necrotizing tracheitis characterized by necrosis (n), squamous metaplasia (s), and hyperplasia of the epithelium and nonsuppurative inflammation (c) in the connective tissue; original magnification ×400. HE stain. C) Control dog at 3 dpi, showing normal alveoli; original magnification ×200. HE stain. D) Influenza-infected dog at 3 dpi, showing severe diffuse necrotizing bronchitis and bronchiolitis with suppurative inflammation in the lumina; original magnification ×100. HE stain. E) Influenza-infected dog at 6 dpi, showing severe necrotizing bronchiolitis; original magnification ×200. HE stain. F) Influenza-infected dog at 6 dpi (serial section of E), showing large amounts of influenza A virus antigens (red stain; arrows) in the bronchiolar epithelium and lumen. Immunohistochemistry; Red Substrate (Dako, Carpinteria, CA, USA); Mayer’s hematoxylin counterstain. G) Influenza-infected dog at 9 dpi, showing severe necrotizing alveolitis with accumulation of necrotic cells in terminal bronchioles (tb) and alveoli (a); original magnification ×200. HE stain.

Gross lesions were limited to the lungs and were characterized by multifocal to coalescing reddish consolidation. In tissues collected on 3, 6 and 9 dpi, histopathologic lesions were observed in the trachea and lungs, and extrapulmonary lesions were absent in puppies infected with the isolate (A/canine/Korea/01/2007 [H3N2]). Severe virus-induced necrosis and inflammation of the upper (trachea and bronchi) and lower (bronchiole and alveoli) respiratory tracts were noted on histologic examination. Although minor differences in the severity of the histologic findings were observed among the 9 infected dogs, all infected dogs shared the following histopathologic features regardless how long after inoculation tissues were collected: 1) moderate to severe multilobular or diffuse necrotizing tracheobronchitis with suppurative inflammation in the lumina and squamous metaplasia of the tracheobronchial epithelium (Figure 3, **panel B**); 2) moderate to severe multilobular or diffuse necrotizing bronchiolitis and alveolitis (i.e., bronchioalveolitis, occasionally accompanied by chronic peribronchiolar and perivascular inflammation) (Figure 3, **panels D** and** E**); and 3) mild to moderate multilobular or diffuse thickening of alveolar septa by infiltrates of inflammatory cells, such as interstitial pulmonary macrophages. At 3, 6, and 9 dpi, large amounts of influenza A virus antigen were found in bronchial and bronchiolar epithelium and lumens (Figure 3, **panel F**).

### Receptor Binding Assay

**Figure 4:**
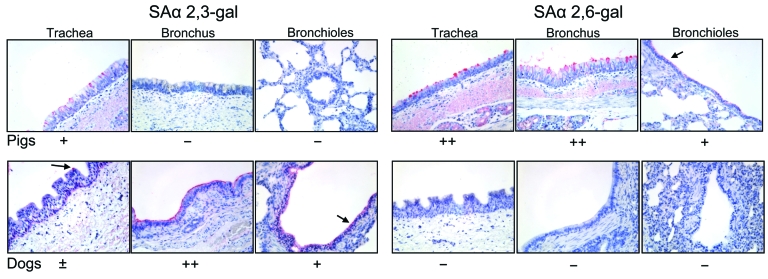
Lectin staining (red stain) for SAα 2,3-gal (avian influenza virus receptors) and SAα 2,6-gal (human influenza virus receptors) in canine trachea, bronchus, and bronchioles, together with porcine tissues as a positive control. Original magnification all x300. −, no staining; ±, rare or few positive cells; +, moderate numbers of positive cells; and ++, many positive cells.

Consistent with the histologic lung lesions, large amounts of SAα 2,3-gal were found on the surface of bronchial and bronchiolar epithelial cells of group NI puppies and were rarely found on tracheal epithelial cells (Figure 4). In contrast, SAα 2,6-gal was not detected in tracheal, bronchial, or bronchiolar epithelial cells, which suggests that canine species may have a lesser role as intermediate hosts for transmission of human influenza viruses to dogs than for avian influenza viruses.

## Discussion

Because all genes of the canine isolates were of avian influenza virus origin, we concluded that the entire genome of the avian influenza virus had been transmitted to the dogs. Transmission of avian influenza A virus to a new mammalian species is of great concern, because it potentially allows the virus to adapt to a new mammalian host, cross new species barriers, and acquire pandemic potential.

Transmission of an entire avian influenza virus to an unrelated mammalian species is a rare event. Several outbreaks of avian influenza infection have occurred in mammals. Influenza virus (H7N7) of avian origin was isolated from the lungs and brains of dead seals. In addition, it was replicated to high titers in ferrets, cats, and pigs and caused conjunctivitis in humans (*11*,*12*). Avian origin influenza virus (H4N5) was reported as the cause of infection and death in harbor seals along the New England coastline (*13*), and avian origin influenza (H5N1) infection was identified in a dog after ingestion of a duck infected with subtype H5N1 during an outbreak in Thailand in 2004 (*14*).

Previously, outbreaks of hemorrhagic pneumonia caused by equine influenza virus (H3N8) were noted in racing dogs, and a human influenza virus (H3N2) was isolated from dogs (*15*,*16*). However, these reports provide limited serologic and virologic evidence for influenza virus infection in dogs. We report the emergence of a new canine influenza virus strain that causes acute respiratory disease in dogs and that differs from previous outbreaks of equine influenza virus (H3N8) infections.

Concerning the possible mechanism of avian influenza virus transmission to dogs, we posit that this transmission results from feeding dogs untreated minced meats of ducks or chickens. In South Korea, untreated duck and chicken meats, including internal organs and heads, have been widely used to feed dogs for fattening in local canine farms or kennels. In a previous study, avian influenza virus (H3N2) was isolated from ducks and chickens sold at live-bird markets in South Korea. Live-bird markets are thought to constitute “a missing link in the epidemiology of avian influenza viruses” because they bring together numerous hosts, such as chickens, ducks, turkeys, geese, and doves, in a high-density setting, which represents an ideal environment for virus interspecies transmission (*17*,*18*). The S11 strain, whose HA and NA genes showed the greatest identity to those of the A/canine/Korea/01/2007 (H3N2) isolates from dogs, was isolated from a tracheal swab of a healthy chicken and is nonpathogenic in poultry (*8*). These observations support the hypothesis that avian influenza virus (H3N2) strains could be transmitted by feeding infected poultry by-products to dogs (*2*).

It is also possible that cross-species transmission of influenza virus occurs directly by aerosol transmission from infected birds to susceptible dogs as a consequence of close contact between the 2 species. Lectin-staining results showed that canine upper (trachea and bronchi) and lower (bronchiole) respiratory tract epithelium cells display SAα 2,3-gal, to which avian influenza viruses bind, making possible a direct transmission of avian influenza viruses from poultry to dogs. Additionally, according to the animal hospital veterinarian, this outbreak was traced to a Jindo dog purchased at a live-animal market in Kyunggi Province that sold chicken, duck, pheasant, rabbit, cats, pet dogs, and other dogs. The Jindo dog was hospitalized at the local animal hospital and may have infected the other pet dogs at the hospital. This epidemiologic result also suggests that the novel canine influenza virus of avian origin was transmitted within canine species.

Antigenic and phylogenetic analyses showed that the HA and NA genes of the A/canine/Korea/01/2007 (H3N2) isolate are closely related to isolates identified in 2003 from chickens and doves in South Korea. Furthermore, HA genes of canine influenza isolates were different from recent isolates from swine in South Korea (*19*). The other genes of the canine influenza isolate are more closely related to those of the subtype H9N2 isolate found in ducks from Hong Kong, the subtype H6N2 isolate from ducks in Japan, and several other avian influenza strains from southeastern China in 2000 through 2005. This finding suggests that multiple variants of subtype H3 influenza viruses may be circulating in these regions and causing disease in pet dogs.

Our experimental reproduction of the disease caused by this isolate induced severe pathologic changes and showed that infected dogs excreted influenza virus (H3N2) in nasal discharge but not in feces. This finding suggests that dog-to-dog transmission of subtype H3N2 could occur through the nasal route and that dog-to-dog transmission of the virus could play an important role in the epizootiology of the disease.

In our study, virologic, serologic, pathologic, and phylogenetic analyses showed cross-species infection of an entire avian influenza A virus (H3N2) to another mammalian species, dogs. Evidence of avian influenza virus infection in pet dogs raises the concern that dogs may be become a new source of transmission of novel influenza viruses, especially where avian influenza viruses are circulating or have been detected.
